# NAVIGATING SOCIAL ISOLATION SCREENING: EXPLORING HEALTH CARE PROVIDER COMFORT FACTORS

**DOI:** 10.1093/geroni/igae098.0671

**Published:** 2024-12-31

**Authors:** Su-I Hou

**Affiliations:** The University of Central Florida, Orlando, Florida, United States

## Abstract

**Purpose:**

This study investigates factors influencing healthcare providers’ comfort levels in screening older patients for social isolation (SI), a significant risk factor for negative health outcomes in this demographic.

**Methods:**

A diverse sample of 59 providers from healthcare and long-term care settings participated. The mean age of providers was 46, 70% female, and 67% white, possessing an average of 17 years of practice experience. The study utilized research-tested scales measuring provider SI screening comfort level, communication about SI, provider-patient interactions (PPI), provider trust in patients (trust), and cultural sensitivity (CS), showing high internal consistencies.

**Results:**

Providers generally scored high on PPI and CS, and moderately on patient trust and comfort with SI screening. Regression analyses considering all factors revealed that providers with higher scores in communication about social isolation (p=.024), patient-provider interaction (p=.022), and those identifying as minority providers (p=.025) demonstrated greater comfort in screening for social isolation (F(6,32)=3.93, p=.005), explaining a substantial 42.4% of the variance. Gender, patient trust, and cultural sensitivity did not reveal a significant impact on the comfort level associated with screening for social isolation (SI).

**Discussion:**

This study suggests that effective communication and interaction, along with demographic factors like minority status, influence providers’ comfort levels in screening for SI. This pilot study demonstrates the reliability of measures, emphasizing the importance of modifiable communication and interaction factors in addressing social determinants of health, such as social isolation in older adults. Further research with a larger sample is recommended.

